# L-shaped association of systemic immune-inflammation index (SII) with serum soluble α-Klotho in the prospective cohort study from the NHANES database

**DOI:** 10.1038/s41598-024-64050-3

**Published:** 2024-06-08

**Authors:** Zujun Wen, Xiang Liu, Tingting Zhang

**Affiliations:** 1 Department of Pharmacy, Heyuan People’s Hospital, Heyuan, China; 2 The First Affiliated Hospital of Chongqing Medical and Pharmaceutical College, Chongqing, China

**Keywords:** Systemic immune-inflammatory index, α-Klotho, Prospective cohort study, L-shaped, NHANES, Immunology, Medical research

## Abstract

The systemic immune-inflammation index (SII), an integrated and ground-breaking inflammatory measure, has been widely used in various fields. We aimed to assess the association between the systemic immune-inflammation index (SII) and α-Klotho (a new anti-aging biomarker). In this cross-sectional investigation, people with complete information on SII and α-Klotho from the National Health and Nutrition Examination Survey (NHANES) between 2007 and 2016 were the study's subject population. SII was calculated by platelet count × neutrophil count/lymphocyte count. The association between SII and α-Klotho was investigated using multivariable linear regression and a generalized additive model. In order to explore the non-linear connection, we employed smoothed curve fitting. Subgroup analysis were also performed. A total of 13,701 participants with an average age of 57.73 ± 10.86 years were enrolled, of whom 51.53% were female. After fully adjustment, SII was negatively associated with serum soluble α-Klotho [β(95% CI) =  − 0.07 (− 0.08, − 0.05)]. Furthermore, we found L-shaped association between SII and klotho protein level, with the inflection point at 255 pg/ml. Subgroup analysis and interaction test revealed that there was no discernible dependence on gender, age, race, smoking, alcohol, diabetes and hypertension (all *p* for interaction > 0.05). SII level was negatively associated with serum klotho protein concentration in American adults. To verify our findings, more large-scale prospective investigations are still required.

## Introduction

The α-Klotho gene, an age-suppressor gene, serves as an obligatory coreceptor for fibroblast growth factor 23 (FGF23) and is abundantly expressed in the distal convoluted tubules of the kidney^1,2^. The α-Klotho primarily appears in two forms: a single-pass transmembrane protein and a soluble protein that can be detected in the blood, urine, and cerebrospinal fluid^1,3^. Klotho, acting as a hormone and being a new anti-aging biomarker, serves to reduce inflammation and oxidative stress^4^. Numerous investigations have demonstrated that transgenic overexpression of α-Klotho increases cardiovascular protection, including improvement of endothelial dysfunction, hypotensive effects, alleviation of cardiac fibrosis, and reduction of arterial stiffness^5–8^, in addition to extending longevity by 20–30%^9^. In contrast, a lack of Klotho can lead to a number of age-related illnesses, including cognitive decline, endothelial dysfunction, reduced bone mineral density, osteoporosis, skin atrophy, and atherosclerosis^10–14^. As the population ages, age-related illnesses are also on the rise, leading to an increase in the disability burden of age-related diseases. In this regard, understanding the elements involved in anti-aging is of great importance.

The systemic immune-inflammation index (SII), calculated by platelet count × neutrophil count/lymphocyte count, is an integrated and ground-breaking inflammatory measure. Developed by Hu et al. in 2014^15^, SII could be an indication of the body's generalized inflammation and local immunological response. Following that, SII was employed to forecast prognosis in a variety of cancers, including cervical cancer^16^, small cell lung cancer^17^, esophageal cancer^18^, epithelial ovarian cancer^19^, and colorectal cancer^20^. In addition, several studies have found that a higher SII is an extremely significant risk factor for nonneoplastic illnesses. According to Ya et al. SII has relevance as a predictor of coronary artery disease (CAD)^21^. And Guo et al. suggested that in those with type 2 diabetes, a higher SII level is linked to diabetic kidney disease^22^. Kaszubowska et al. found that a positive correlation between blood Klotho levels and lymphocyte secretion, which may be connected to Klotho's capacity to preserve immunological homeostasis. Furthermore, it has been demonstrated that Klotho has a protective role throughout the inflammatory process, and platelet volume is thought to positively correlate with serum Klotho concentrations. SII, offering a more thorough assessment of the systemic immune-inflammatory condition by combining many factors (platelet counts, lymphocyte counts, and neutrophil counts), should be more potential to predict aging pathways. However, few research has examined the relationship between SII and aging in the general old population, and it is still unclear if SII can be used to predict aging.

As a result, the purpose of our study was to assess the association between the systemic immune-inflammation index (SII) and α-Klotho (a new anti-aging biomarker) among the National Health and Nutrition Examination Survey (NHANES) participants in the US. We hypothesized that an elevated SII would be associated with a lower klotho protein level.

## Materials and methods

### Data source

The data for this study was collected from NHANES, a national population-based cross-sectional survey that was carried out by the National Center for Health Statistics (NCHS) to assess more about the nutrition status and potential health risk factors of non-institutionalized US civilians. To get a representative sample of the whole American population, a sophisticated stratified, multistage probability cluster sampling design was created^23^. Participants were interviewed at home to get information on their socioeconomic standing, health, and other aspects. Lab and physical examinations were conducted in a mobile examination facility.

The protocols for the NHANES study were approved by the NCHS's Research Ethics Review Board. All participants in the survey provided their written informed permission, or in the case of individuals under the age of 16, the consent of a parent or legal guardian. All specific NHANES research designs and data are available to the public on the website www.cdc.gov/nchs/nhanes/. This cross-sectional study adhered to the STROBE (Strengthening the Reporting of Observational Studies in Epidemiology) reporting standards^24^.

### Study population

**Figure 1:**
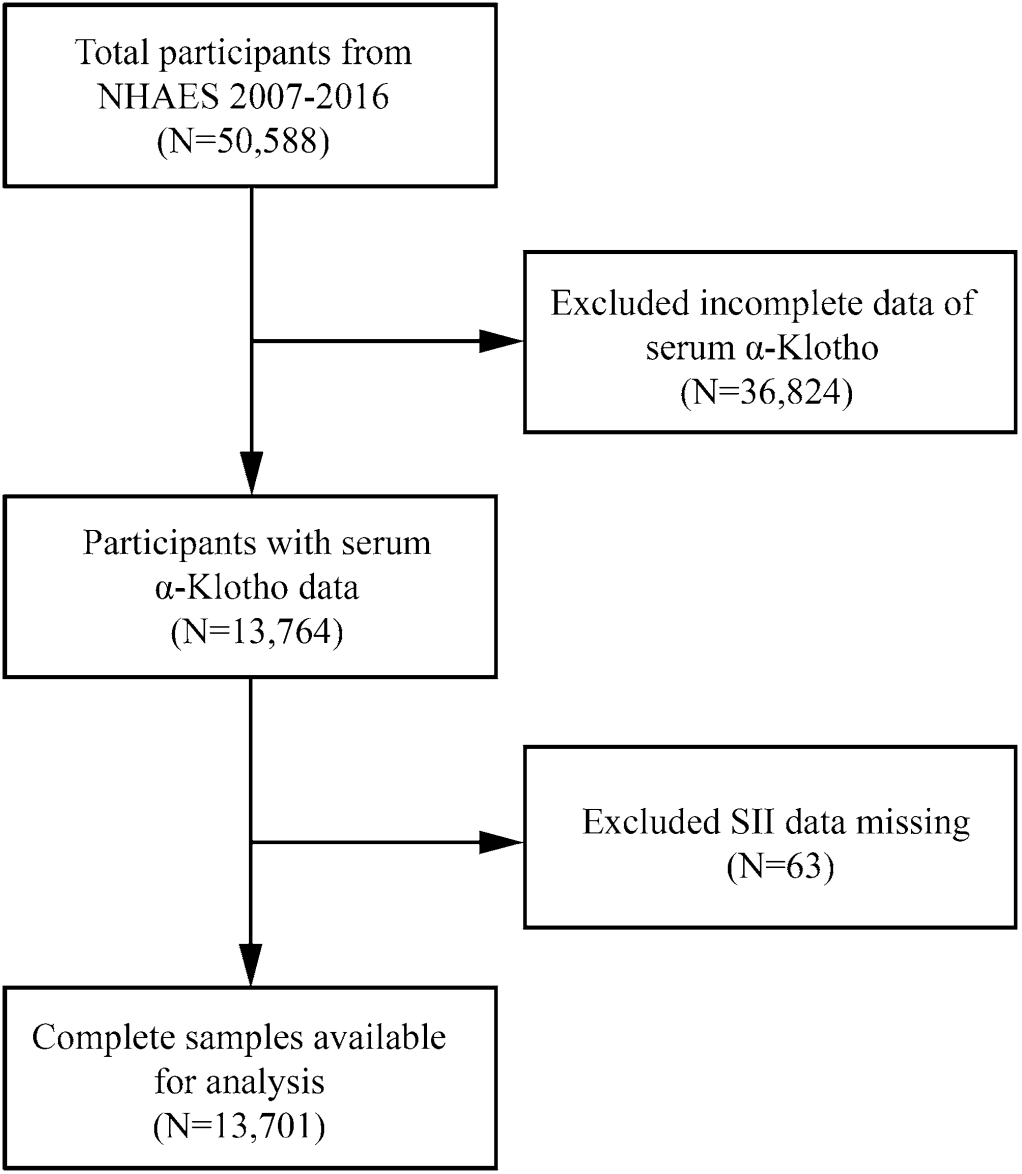
Flowchart of the sample selection from NHANES 2007–2016. A total of 50,588 participants were enrolled. After excluding those lacking data about the serum klotho protein (n = 36,824) and the SII information (n = 63), 13,701 eligible participants were included in our final analysis.

Data from five National Health and Nutrition Examination Survey (NHANES) cycles (NHANES 2007–2008, 2009–2010, 2011–2012, 2013–2014, and 2015–2016 cycle) were analyzed in the research design, since klotho protein was only included during that time. Participants who had comprehensive information regarding the klotho protein and SII were included in our analysis. Initially, a total of 50,588 participants were enrolled. After excluding those lacking data about the serum klotho protein (n = 36,824) and the SII information (n = 63), 13,701 eligible participants were included in our final analysis (Fig. 1).

### Definition of systemic immune-inflammation index

Complete blood counts were employed to calculate the lymphocyte, neutrophil, and platelet counts, which were recorded as 10^3^ cells/μL using automatic hematology analysis equipment (Coulter DxH analyzer 800). SII was calculated as plate count × neutrophil count/lymphocyte count as previous studies reported^15,25^. Participants were divided into groups based on their SII quartile for subsequent analysis, and SII was also used as a continuous variable in the analysis. In our study, SII was intended as an exposure variable.

### Assessment of α-Klotho

Blood samples were collected from participants aged 40–79 years during the five cycles of the NHANES project, stored in dry ice at a temperature of − 80 °C at the Centers for Disease Control and Prevention (CDC) in Atlanta, Georgia, and sent to the Northwest Lipid Metabolism and Diabetes Research Laboratory at the University of Washington in Seattle, Washington. Serum Klotho levels in the fresh-frozen samples were tested by the Human Soluble α-Klotho Assay Kit. Every sample was examined twice, and the average of the findings served as the resulting value. The regional supervisor can evaluate the analytical results once they have been automatically transmitted from the device to the lab's Oracle management system. Reproducible analyses are those that produce repeated findings for the samples of 10% or higher. If the value of the quality control sample did not fall within two standard deviations (SDs) of the target value, the analysis was deemed invalid and the sample analysis was redone. α-Klotho was intended as an outcome variable in our research.

### Assessment of covariates of interest

Potential confounders were selected a priori according to a literature review^26,27^. So our investigation took into account other covariates, such as gender (men/women), age (years), race (Mexican American/Other Hispanic/Non-Hispanic White/Non-Hispanic Black/Other Race—Including Multi-Racial), marital status (widowed/divorced/separated/never married/living with partner), education level (less than 9th grade/9-11th grade/high school graduate/GED or equivalent/some college or AA degree/college graduate or above), smoking (≥ 100 cigarettes in life/ < 100 cigarettes in life), alcohol (≥ 12 drinks/year/ < 12 drinks/year), diabetes (yes/no) and hypertension (yes/no), which may affect the association between SII and α-Klotho. Diabetes (yes/no) and hypertension (yes/no) are defined with the questionnaire. Public access to the detailed measurement techniques for these variables is available at www.cdc.gov/nchs/nhanes/.

### Statistical analysis

Using proper NHANES sample weights and taking into consideration the complicated multistage cluster survey, all statistical analysis was carried out in accordance with the Centers for Disease Control and Prevention's (CDC) recommendations.

Means with standard errors (SE) were used to summarize continuous variables, while proportions were used to present categorical characteristics. The differences among individuals were assessed using weighted one-way analysis of variance (ANOVA) or a weighted chi-square test. In a typical multivariate regression analysis, the goal is to understand the relationship between a dependent variable (also known as the outcome or response variable) and one or more independent variables (also known as predictors or explanatory variables), while controlling for the effects of other variables. Taking into account the complicated sampling design (sampling weights) of the NHANES, the association between SII and serum soluble α-Klotho was examined using multivariable regression models, which allow for the control of multiple covariates simultaneously. In model 1, covariates were not adjusted at all. In model 2, gender, age and race were adjusted. Model 3 were adjustments for gender, age, race, marital status, education level, smoking status, alcohol, diabetes and hypertension. In these model, α-Klotho was intended as the dependent variable, while SII and SII quartile were intended as the independent variables. Stratified multivariable linear regression models with stratified covariates such as gender (men/women), age (years), race (Mexican American/Other Hispanic/Non-Hispanic White/Non-Hispanic Black/Other Race—Including Multi-Racial), smoking (≥ 100 cigarettes in life/ < 100 cigarettes in life), alcohol (≥ 12 drinks/year/ < 12 drinks/year), diabetes (yes/no) and hypertension (yes/no). The SII was converted into a continuous variable for sensitivity analysis to evaluate its robustness. Then we employed a generalized additive model (GAM) and smooth curve fitted to address the non-linearity of SII with α-Klotho and each stratification. A two-piecewise linear regression model (segmented regression model) was used to fit each period and determine the threshold effect if a non-linear connection was shown. If a non-linear connection was discovered, the threshold effect was determined by fitting each interval with a segmented regression model, also known as a two-piecewise linear regression model. Based on the model that produces the highest likelihood, the breakpoint (K) connecting the segments was discovered using the two-step recursive approach.

All analyses were performed using R version 3.4.3 (http://www.R-project.org, The R Foundation) and Empower software (www.empowerstats.com; X&Y solutions, Inc., Boston, MA). *p* < 0.05 was considered as the statistically significant level. The *p* value for interaction represented interaction between the covariate and the exposure of interest on the outcome.

### Ethics statement

The studies involving human participants were reviewed and approved by the NCHS research ethics review board. The patients/participants provided their written informed consent to participate in NHANES.

## Results

### Baseline characteristics of study participants

Based on the SII quartile, the research participants’ characteristics are shown in Table 1. A total of 13,701 participants with an average age of 57.73 ± 10.86 years were enrolled, of whom 51.53% were female. Among the SII Q1-Q4 groups, differences with statistical significance were identified in gender, race, marital status, education level, smoking status, alcohol, diabetes, hypertension, BMI and klotho (all *p* < 0.05). In comparison to the Q1, Q2 and Q3 (Q1: 885.70 ± 335.79 pg/ml, Q2: 853.04 ± 303.99 pg/ml, *p* < 0.001 and Q3: 846.06 ± 276.31 pg/ml, *p* < 0.001), the serum klotho protein concentration was lowest in Q4 (Q4: 813.35 ± 272.22 pg/ml).

**Table 1:** Baseline characteristics of study participants by the systemic immune-inflammation index (SII) quartile in NHANES 2007–2016.

Characteristic	SII levels				*p* value
	Quartile 1 (≤ 343.63)	Quartile 2 (343.63–460)	Quartile 3 (460–645.65)	Quartile 4 (> 645.65)	
Age (%)					0.013
40–59	65.05	65.38	66.21	64.93	
60–69	22.59	33.97	20.79	20.83	
70–79	12.36	11.66	13.00	14.24	
Gender (%)					< 0.001
Men	52.02	50.38	47.34	44.88	
Women	47.98	49.62	52.66	55.12	
Race (%)					< 0.001
Mexican American	6.11	7.64	6.61	5.85	
Other Hispanic	4.51	4.36	3.85	3.68	
Non-Hispanic White	66.92	74.22	76.23	79.11	
Non-Hispanic Black	17.36	9.29	7.57	6.06	
Other Race—Including Multi-Racial	5.11	4.49	5.74	5.30	
Education level (%)					< 0.001
Less than 9th grade	7.00	7.85	7.88	6.79	
9–11th grade	12.76	9.72	12.51	14.28	
High school graduate/GED or equivalent	24.04	25.11	23.03	24.40	
Some college or AA degree	27.20	28.67	27.82	28.33	
College graduate or above	29.01	28.92	28.76	26.19	
Marital status (%)					< 0.001
Married	65.76	70.88	67.33	63.98	
Widowed	5.65	5.27	6.49	7.14	
Divorced	13.67	12.33	13.80	14.78	
Separated	2.34	2.16	2.27	3.21	
Never married	8.01	4.89	6.08	7.12	
Living with partner	4.58	4.47	4.03	3.77	
Smoking (%)					0.006
≥ 100 cigarettes in life	47.67	48.34	50.01	51.62	
< 100 cigarettes in life	52.33	51.66	49.99	48.38	
Alcohol (%)					< 0.001
≥ 12 drinks/year	69.50	70.73	73.68	68.04	
< 12 drinks/year	30.50	29.27	26.32	31.96	
Diabetes (%)					< 0.001
Yes	11.55	10.76	10.75	14.21	
No	88.45	89.24	89.25	85.79	
Hypertension (%)					0.003
Yes	39.64	40.08	39.27	43.17	
No	60.36	59.92	60.73	56.83	
Body mass index (kg/m^2^)	28.47 ± 5.77	29.34 ± 6.20	29.09 ± 6.17	29.83 ± 7.27	< 0.001
Neutrophils count (10^3^/μL)	2.90 ± 0.97	3.67 ± 0.96	4.34 ± 1.14	5.58 ± 1.74	< 0.001
Lymphocyte count (10^3^/μL)	2.40 ± 1.06	2.20 ± 0.68	2.04 ± 0.62	1.81 ± 0.60	< 0.001
Platelet count (10^3^/μL)	205.79 ± 50.33	237.82 ± 51.32	257.83 ± 52.88	297.99 ± 74.53	< 0.001
Neutrophil to lymphocyte ratio	1.28 ± 0.38	1.72 ± 0.38	2.21 ± 0.50	3.29 ± 1.26	< 0.001
Klotho (pg/ml)	885.70 ± 335.79	853.04 ± 303.99	846.06 ± 276.3	813.35 ± 272.22	< 0.001

### The association between systemic immune-inflammation index (SII) with serum soluble α-Klotho

The association between SII and α-klotho was demonstrated in Table 2. Our study indicated that SII had a negative association with α-klotho in the crude model [β(95% CI) =  − 0.08 (− 0.10, − 0.07)], minimally adjusted model [β(95% CI) =  − 0.08 (− 0.10, − 0.07)], and the fully adjusted model [β(95% CI) =  − 0.07 (− 0.08, − 0.05)]. This correlation retained its statistical significance when SII was categorized as quartiles. We found that with the increase in SII levels, the β values of the Q2 to Q4 groups showed a downwards trend. In addition, comparing to participants in the Q1 group, individuals in the Q4 group had a significantly lower klotho protein level in model 3 [β(95% CI) =  − 67.65 (− 82.84, − 52.45)]. And all *p* values for trend were less than 0.001.

**Table 2:** Association between immune-inflammation index (SII) and serum anti-aging protein klotho.

	β (95% CI), *p* value		
	Crude model	Minimally adjusted model	Fully adjusted model
	(Model 1)	(Model 2)	(Model 3)
Continuous	− 0.08 (− 0.10, − 0.07) < 0.001	− 0.08 (− 0.10, − 0.07) < 0.001	− 0.07 (− 0.08, − 0.05) < 0.001

Quartile 1	Reference	Reference	Reference
SII quartile			
Quartile 2	− 34.44 (− 49.06, − 19.82) < 0.001	− 30.37 (− 44.97, − 15.77) < 0.001	− 30.39 (− 45.28, − 15.50) 0.179
Quartile 3	− 55.44 (− 70.06, − 40.82) < 0.001	− 50.40 (− 65.05, − 35.74) < 0.001	− 48.76 (− 63.73, − 33.79) < 0.001
Quartile 4	− 78.78 (− 93.40, − 64.16) < 0.001	− 70.62 (− 85.38, − 55.86) < 0.001	− 67.65 (− 82.84, − 52.45) < 0.0001
*p* for trend	< 0.001	< 0.001	< 0.001

All estimated results were expressed as β (95% CI); β, Partial regression coefficient; CI, Confidence interval.

Model 1: Covariates were not adjusted at all.

Model 2: Adjusted for gender, age, and race.

Model 3: Adjusted for gender, age, race, marital status, education level, smoking status, alcohol, diabetes and hypertension.

### Non-linear analysis

**Figure 2:**
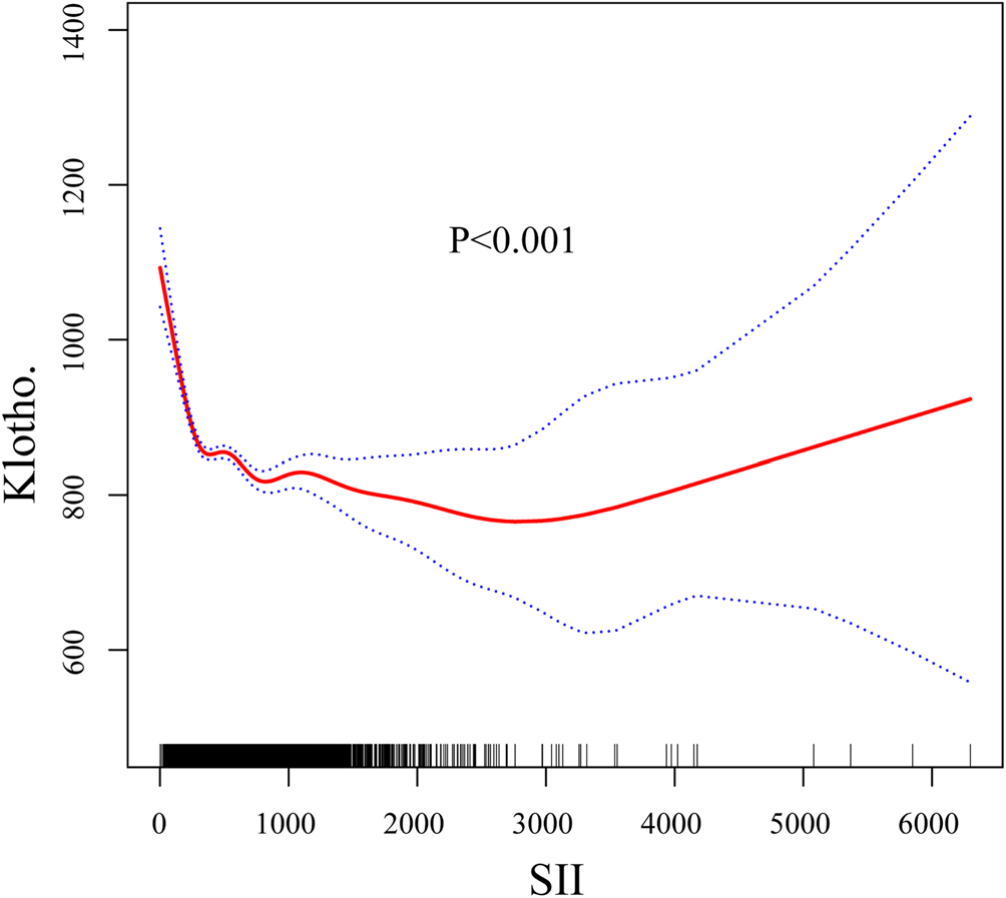
Smooth curve fitting for SII and klotho.

To investigate the non-linear association between SII and serum klotho protein concentration, we conducted a smooth curve fitting and a segmented regression (Fig. 2; Table 3). The smooth curve after fully adjustment revealed the L-shaped association between SII and klotho protein level. The inflection point was 255 pg/ml. The two-piecewise linear regression models indicated that the serum klotho protein concentration gradually declined as the SII level increased (SII < 255 pg/ml), while there was no discernible decreasing trend when SII > 255 pg/ml.

**Table 3:** Threshold effect analysis for the relationship between immune-inflammation index (SII) and serum anti-aging protein klotho in NHANES 2007–2016.

Model	Serum klotho protein concentration Adjusted β (95% CI), *p* value
Fitting by standard linear model	− 0.07 (− 0.08, − 0.05), < 0.001
Fitting by two-piecewise linear model	
Breakpoint (K)	255
β1 (< K)	− 1.08 (− 1.30, − 0.87), < 0.001
β2 (> K)	− 0.04 (− 0.06, − 0.03), < 0.001
β2/β1	1.04 (0.82, 1.26), < 0.001
Logarithmic likelihood ratio test *p* value	< 0.001

Adjusted for gender, age, race, marital status, education level, smoking status, alcohol, diabetes and hypertension.

### Subgroup analysis

Subgroup analysis was subsequently employed to examine if the association between SII and serum klotho protein concentration was stable across a range of demographic situations. Our research revealed that there was no dependence on the association between SII and serum klotho protein concentration. As can be observed in Table 4, none of the stratifications, including age, gender, race, smoking, alcohol, diabetes and hypertension significantly impacted the negative association between SII and serum klotho protein concentration (all *p* for interaction > 0.05). In our study, all *p* values for interaction were greater than 0.05, suggesting that there is no effect measure modification present for any of the covariates considered.

**Table 4:** Subgroups analyses of the effect of SII on serum anti-aging protein klotho.

Subgroups	N	β (95% CI), *p* value	*p* for interaction
Age			0.5669
40–59	7442	− 0.06 (− 0.08, − 0.04), < 0.001	
60–69	3833	− 0.07 (− 0.10, − 0.04), < 0.001	
70–79	2426	− 0.08 (− 0.11, − 0.05), < 0.001	
Gender			0.4890
Men	6641	− 0.06 (− 0.08, − 0.04), < 0.001	
Women	7060	− 0.07 (− 0.10, − 0.05), < 0.001	
Race			0.4282
Mexican American	2180	− 0.08 (− 0.12, − 0.04), < 0.001	
Other Hispanic	1571	− 0.11 (− 0.16, − 0.05), < 0.001	
Non-Hispanic White	5901	− 0.06 (− 0.09, − 0.04), < 0.001	
Non-Hispanic Black	2702	− 0.06 (− 0.10, − 0.02), 0.0029	
Other Race—Including Multi-Racial	1347	− 0.06 (− 0.10, − 0.02), 0.1041	
Smoking			0.1978
≥ 100 cigarettes in life	6648	− 0.06 (− 0.08, − 0.04), < 0.001	
< 100 cigarettes in life	7053	− 0.08 (− 0.10, − 0.06), < 0.001	
Alcohol			0.5776
≥ 12 drinks/year	8976	− 0.06 (− 0.08, − 0.04), < 0.001	
< 12 drinks/year	4725	− 0.07 (− 0.10, − 0.05), < 0.001	
Diabetes			0.2736
Yes	2462	− 0.09 (− 0.12, − 0.05), < 0.001	
No	11,239	− 0.06 (− 0.08, − 0.05), < 0.001	
Hypertension			0.4170
Yes	6365	− 0.07 (− 0.10, − 0.05) < 0.001	
No	7336	− 0.06 (− 0.08, − 0.04) < 0.001	

Subgroup analysis for the association between SII and serum anti-aging protein klotho. None of the stratifications including gender, age, race, marital status, education level, smoking status, alcohol, diabetes and hypertension affected the association of SII and klotho.

## Discussion

In this study, the association of systemic immune-inflammation index (SII) with serum soluble α-klotho in non-institutionalized Americans was assessed. After fully adjustment, we identified L-shaped correlations between SII and klotho protein level, suggesting that lower SII levels were substantially linked with higher serum klotho protein concentration within a certain range. The results of a subgroup analysis and an interaction test demonstrated that the association between SII and serum klotho protein concentration was stable across various demographic situations.

The association between α-klotho and inflammatory cytokines has not been extensively studied. Martín-Núñez et al. found that Patients with atherosclerotic vascular disease had lower blood concentrations and vascular expression of Klotho than control participants, although their inflammatory state was much greater, suggesting a negative and significant correlation between inflammatory parameters (*TNF-α*,* IL-6*, and *IL-10*) and Klotho^11^. In a cross-sectional analysis, it was shown that s-Klotho was weakly negatively linked with CRP levels in elderly populations (> 65 years) (R: − 0.245; *p* = 0.022)^28^. However, rather than studying the whole populace, these investigations were carried out in certain groups (based either on disease history or age). This study investigated the relationship between aging and inflammation by looking at the relationship between the Systemic Immune Inflammatory Index (SII) and serum soluble alpha-klotho in community-dwelling Americans.

To the best of our knowledge, this is the first study to examine the link between SII and α-klotho, emphasizing how a lower SSI level is associated with serum klotho protein concentration. The systemic immune-inflammation index (SII), integrated platelet, neutrophil, and lymphocyte kinds of inflammation cells, has been considered as a stable index to reflect the local immune response and systemic inflammation^15^. As an inflammatory marker, SII has been widely used in various fields. According to Tang et al., SII may be a useful and practical inflammatory marker that may be used to forecast the likelihood of low bone mineral density or osteoporosis in postmenopausal women over the age of 50^29^. And Qin et al. found that A higher SII level was independently linked to an increased risk of albuminuria (OR = 1.31; 95% CI 1.17–1.48, *p* < 0.0001)^30^. Moreover, Xie et al. uncovered that SII levels had a positive connection with hepatic steatosis but no discernible link with liver fibrosis^31^. Additionally, the SII has been confirmed to be more reliable and accurate at predicting outcomes than other traditional inflammatory factors^32,33^. In summary, SII has demonstrated exceptional predictive power in a number of investigations. At the same time, SII was regarded as an excellent inflammatory marker in clinical practice with widely clinical applications by a non-invasive approach, simple access, and low cost. Based on the findings of this investigation, we put forth the hypothesis that, in the event that the concentrations of SII and Klotho are causally related, then increasing endogenous Klotho protein secretion or supplying Klotho protein externally could have a major positive clinical impact on the management of inflammation.

The precise mechanism behind the correlation between inflammation and klotho is currently unknown. Moreno JA et al. displayed the expression of Klotho is downregulated by inflammatory cytokines including TWEAK and TNF via the transcription factor nuclear factor-kappaB (NFκB) dependent mechanism^34^. Wu SE et al. suggested that lower s-Klotho levels may be linked to inflammation, particularly in clinical conditions where mean platelet volume (MPV) is decreased and white blood cell (WBC) count is increased^35^. In disorders of the heart and kidney, s-Klotho may play a greater role as an inverse signal of inflammation. In cardiovascular disease, Klotho inhibits TRPC6 channels in cardiomyocytes^36^, decreases IL-6 production in endothelial cells^37^, and deactivates ROS and NF-B-mediated inflammation^38^. Through the suppression of protein kinase-1/2, p38 mitogen-activated protein kinase, and insulin-like growth factor 1, Klotho lessens podocyte damage in the kidneys^39^.

We must admit that our study has certain restrictions. First, due to the fact that this research is cross-sectional analysis, temporality cannot be determined. Furthermore, despite controlling for a number of pertinent confounders, we were unable to completely exclude the influence of other confounding variables, thus our results should be adopted with care. Additionally, it is yet uncertain if our findings apply to other countries because they were based on just one. Therefore, more prospective cohort studies or Mendelian randomized trials in other populations are required to corroborate the finding of causation in the future.

In despite of these limitations, there are a number of benefits to our research. This is the first investigation into the relationship between SII and -klotho, revealing how a lower SSI level is linked to a higher quantity of serum klotho protein. Our study is typical of the multiethnic and gender-diverse adult population in the United States since we employed a nationally representative sample. Due to the high sample size, we utilized subgroup analyses on all covariates to confirm the reliability of the regression analysis after correcting for confounders.

## Conclusion

Our research discovered that the SII level was independently and negatively associated with serum klotho protein concentration. To verify our findings, more large-scale prospective investigations are still required.

## Data Availability

Publicly available datasets were analyzed in this study. This data can be found here: www.cdc.gov/nchs/nhanes/.
